# Supplementary material for: Knowledge and practices regarding diabetic retinopathy among diabetic patients registered in a chronic disease management system in eastern China
Source: PLoS One. 2020 Aug 21;15(8):e0234733. doi: 10.1371/journal.pone.0234733 (PMC7444505; doi:10.1371/journal.pone.0234733)
Supplement: Attachment — Submitted filename: PONE-D-19-29504_reviewer VG.pdf [file pone.0234733.s001.pdf]

PLOS ONE

Knowledge and attitudes regarding diabetic retinopathy among diabetic patients registered in a chronic disease management system in eastern China

--Manuscript Draft--

**Manuscript Number:**	PONE-D-19-29504
**Article Type:**	Research Article
**Full Title:**	Knowledge and attitudes regarding diabetic retinopathy among diabetic patients registered in a chronic disease management system in eastern China
**Short Title:**	Knowledge regarding diabetic retinopathy among diabetic patients in Chinese chronic disease management system
**Corresponding Author:**	Xiang Chen<br>Sun Yat-Sen University Zhongshan Ophthalmic Center<br>Guangzhou, CHINA
**Keywords:**	
**Abstract:**	Purpose<br>To investigate the knowledge and attitudes regarding diabetic retinopathy (DR) among diabetic patients included in acommunity-based primary health system (CBPHS) in China.<br>Methods<br>Diabetic patientsaged 18 years and aboveregistered in the CBPHS in Yueqing city, Zhejiang province were recruited. Information obtained by questionnaire included: demographic and socioeconomic status, knowledge about DR, and ocular and medical history. The primary outcome was whether the participant knew that DM can affect the eyes, defined according to the question: “Do you know diabetes mellitus (DM) can affect eyes? (yes or no)”. A knowledge score was calculated based on the responses to seven questions, with 1 point awarded for a correct response and 0 points for an incorrect or uncertain answer. Results<br>A total of 1972 diabetic patients were included in the study with an average age of 65.2±10.8 years, 45.7% were male. ~~The~~ 1219 patients (61.8%) knew that DM can affect the eyes. Significant differences in age, education, income status, ~~the~~ insurance covering eye care, fasting blood glucose, duration of DM, history of hypertension existed between subjects who knew and those who did not know that DM can affect the eyes (P<0.05 for all). The proportion of correct answers to the DR knowledge questions ranged from 33.3% to 61.8%, with an average score of 3.65±2.47.In the multiple regression analysis, the knowledge score was significantly associated with age, education, income, history of hypertension, duration of DM, being told that regular examinations should be performed and concern about vision loss (P <0.01 for all).<br>Conclusions<br>The knowledge and attitudes toward DR among DM patients were still low within the chronic disease management system in eastern China. Routine ophthalmic screening, health care promotions, and educational programs should be emphasized and implemented for better DR prevention and management.
**Order of Authors:**	Fang Duan
	Yan Zheng
	Qian Zhao
	Ze Huang
	Yuedan Wu
	Guoyi Zhou
	Xiang Chen
**Additional Information:**	
**Question**	**Response**

Powered by Editorial Manager® and ProduXion Manager® from Aries Systems Corporation

**Financial Disclosure**<br><br>Enter a financial disclosure statement that describes the sources of funding for the work included in this submission. Review the submission guidelines for detailed requirements. View published research articles from *PLOS ONE* for specific examples.<br><br>This statement is required for submission and **will appear in the published article** if the submission is accepted. Please make sure it is accurate.<br><br>**Unfunded studies**<br>Enter: *The author(s) received no specific funding for this work.*<br><br>**Funded studies**<br>Enter a statement with the following details:<br>• Initials of the authors who received each award<br>• Grant numbers awarded to each author<br>• The full name of each funder<br>• URL of each funder website<br>• Did the sponsors or funders play any role in the study design, data collection and analysis, decision to publish, or preparation of the manuscript?<br>• **NO** - Include this sentence at the end of your statement: *The funders had no role in study design, data collection and analysis, decision to publish, or preparation of the manuscript.*<br>• **YES** - Specify the role(s) played.<br><br>* typeset	The authors received no specific funding for this work.
**Competing Interests**<br><br>Use the instructions below to enter a competing interest statement for this submission. On behalf of all authors, disclose any competing interests that could be perceived to bias this work—acknowledging all financial support and any other relevant financial or non-financial competing interests.<br><br>This statement **will appear in the published article** if the submission is accepted. Please make sure it is	The authors declare no competing interests.

accurate. View published research articles from *PLOS ONE* for specific examples.<br><br>**NO authors have competing interests**<br><br>Enter: *The authors have declared that no competing interests exist.*<br><br>**Authors with competing interests**<br><br>Enter competing interest details beginning with this statement:<br><br>*I have read the journal's policy and the authors of this manuscript have the following competing interests: [insert competing interests here]*<br><br>* typeset	
**Ethics Statement**<br><br>Enter an ethics statement for this submission. This statement is required if the study involved:<br><br>• Human participants<br>• Human specimens or tissue<br>• Vertebrate animals or cephalopods<br>• Vertebrate embryos or tissues<br>• Field research<br><br>Write "N/A" if the submission does not require an ethics statement.<br><br>General guidance is provided below. Consult the submission guidelines for detailed instructions. **Make sure that all information entered here is included in the Methods section of the manuscript.**	Ethical approval was obtained from the Yueqing Hospital Ethical Review Board (YQYY201700001), and the study adhered to the Declaration of Helsinki. Written informed consent was received from all participants.

Powered by Editorial Manager® and ProduXion Manager® from Aries Systems Corporation

**Format for specific study types**<br><br>**Human Subject Research (involving human participants and/or tissue)**<br>• Give the name of the institutional review board or ethics committee that approved the study<br>• Include the approval number and/or a statement indicating approval of this research<br>• Indicate the form of consent obtained (written/oral) or the reason that consent was not obtained (e.g. the data were analyzed anonymously)<br><br>**Animal Research (involving vertebrate animals, embryos or tissues)**<br>• Provide the name of the Institutional Animal Care and Use Committee (IACUC) or other relevant ethics board that reviewed the study protocol, and indicate whether they approved this research or granted a formal waiver of ethical approval<br>• Include an approval number if one was obtained<br>• If the study involved *non-human primates*, add *additional details* about animal welfare and steps taken to ameliorate suffering<br>• If anesthesia, euthanasia, or any kind of animal sacrifice is part of the study, include briefly which substances and/or methods were applied<br><br>**Field Research**<br><br>Include the following details if this study involves the collection of plant, animal, or other materials from a natural setting:<br>• Field permit number<br>• Name of the institution or relevant body that granted permission	
**Data Availability**<br><br>Authors are required to make all data underlying the findings described fully available, without restriction, and from the time of publication. PLOS allows rare exceptions to address legal and ethical concerns. See the PLOS Data Policy and FAQ for detailed information.	Yes - all data are fully available without restriction

A Data Availability Statement describing where the data can be found is required at submission. Your answers to this question constitute the Data Availability Statement and **will be published in the article**, if accepted.<br><br>**Important:** Stating 'data available on request from the author' is not sufficient. If your data are only available upon request, select 'No' for the first question and explain your exceptional situation in the text box.<br><br>Do the authors confirm that all data underlying the findings described in their manuscript are fully available without restriction?	
**Describe where the data may be found in full sentences. If you are copying our sample text, replace any instances of XXX with the appropriate details.**<br><br>• If the data are **held or will be held in a public repository**, include URLs, accession numbers or DOIs. If this information will only be available after acceptance, indicate this by ticking the box below. For example: *All XXX files are available from the XXX database (accession number(s) XXX, XXX.*).<br>• If the data are all contained **within the manuscript and/or Supporting Information files**, enter the following: *All relevant data are within the manuscript and its Supporting Information files.*<br>• If neither of these applies but you are able to provide **details of access elsewhere**, with or without limitations, please do so. For example:<br><br>*Data cannot be shared publicly because of [XXX]. Data are available from the XXX Institutional Data Access / Ethics Committee (contact via XXX) for researchers who meet the criteria for access to confidential data.*<br><br>*The data underlying the results presented in the study are available from (include the name of the third party*	All relevant data within the manuscript and its supporting information.

and contact information or URL).<br>• This text is appropriate if the data are owned by a third party and authors do not have permission to share the data.<br><br>* typeset	
Additional data availability information:	

Manuscript Click here to download Manuscript DF_DR_plos one.docx

Knowledge and attitudes regarding diabetic retinopathy among diabetic patients registered in a chronic disease management system in eastern China

Fang Duan[1#], Yan Zheng[2#], Qian Zhao[2], Ze Huang[2], Yuedan Wu[2], Guoyi Zhou[2*], Xiang chen[1*]

Zhongshan Ophthalmic Center, State Key Laboratory of Ophthalmology, Sun Yat-sen University, 54 Xianlie Road, Guangzhou 510060, China

Affiliated Yueqing Hospital, Wenzhou Medical University, 338 Qingyuan Road, Wenzhou, Zhejiang, 325600, China

*** Corresponding Authors**

Email: chen1094@hotmail.com(XC)

Email: zhougy123456@163.com(GZ)

#These authors contributed equally to this work.

ABSTRACT

Purpose

To investigate the knowledge and attitudes regarding diabetic retinopathy (DR) among diabetic patients included in a community-based primary health system (CBPHS) in China.

Methods

Diabetic patients aged 18 years and above registered in the CBPHS in Yueqing city, Zhejiang province were recruited. Information obtained by questionnaire included: demographic and socioeconomic status, knowledge about DR, and ocular and medical history. The primary outcome was whether the participant knew that DM can affect the eyes, defined according to the question: "Do you know diabetes mellitus (DM) can affect eyes? (yes or no)". A knowledge score was calculated based on the responses to seven questions, with 1 point awarded for a correct response and 0 points for an incorrect or uncertain answer.

Results

A total of 1972 diabetic patients were included in the study with an average age of 65.2±10.8 years, 45.7% were male. ~~The~~ 1219 patients (61.8%) knew that DM can affect the eyes. Significant differences in age, education, income status, ~~the~~ insurance covering eye care, fasting blood glucose, duration of DM, history of hypertension existed between subjects who knew and those who did not know that DM can affect the eyes ($P<0.05$ for all). The proportion of correct answers to the DR knowledge questions ranged from 33.3% to 61.8%, with an average score of 3.65±2.47. In the multiple regression analysis, the knowledge score was significantly associated with age, education, income, history of hypertension, duration of DM, being told that regular examinations should be performed and concern about vision loss ($P <0.01$ for all).

Conclusions

The knowledge and attitudes toward DR among DM patients were still low within the chronic disease management system in eastern China. Routine ophthalmic screening, health care promotions, and educational programs should be emphasized and implemented for better DR prevention and management.

Introduction

Diabetes mellitus (DM) is a major public health problem worldwide, and its prevalence is increasing at an alarming rate with population growth and aging[1]. As reported by the International Diabetes Federation (IDF), there were 451 million diabetic patients worldwide in 2017, which is expected to increase to 693 million by 2045[2]. It was reported that the prevalence of DM in China reached 10.9% in 2013, which was nearly 10-fold of that in the 1980s, and that for prediabetes was 35.7%[3]. Diabetic retinopathy (DR) is a leading cause of vision loss worldwide and a common ocular complication of DM, occurring in one of three diabetic patients[4-7]. However, previous studies have found that nearly half of the DR patients in China have never received any ocular examination[8].

Early detection and intervention of DR had been showed to be critical to prevent irreversible blindness and improve the patient's quality of life[9]. And the efficacy and cost effectiveness of early detection and treatment of DR had been well established[10]. Previous studies found that a lack of DR knowledge was associated with poor patients adherence[8, 11]. In our previous study, we used an in-depth interview method to explore the influencing factors on the compliance of timely visits among patients with proliferative diabetic retinopathy, and found that more than 90% of the patients were lacking knowledge about DR[12]. A recent Cochrane review showed that interventions to increase awareness about DR are vital in improving attendance for DR screening, and thus a potentially important solution for reducing blindness caused by DR[13]. Therefore patient awareness and knowledge about DR will be the key to successful disease management and prevention.

In order to support patients with chronic diseases, a community-based primary health system (CBPHS) was introduced throughout the entire nation to enable access to basic and less costly healthcare services, especially after the New Health Reform in 2009, which called for medical insurance coverage for more than 90% of Chinese people[14]. Hospital-based model has been shifted to delivery in primary settings. The CBPHS has proven to be very helpful to manage chronic diseases[15]. Free regular blood glucose

tests are provided for diabetes patients registered in the CBPHS, but DM complications are not necessarily assessed. Since the previous studies on the patient awareness and knowledge of DR were based on general population, and the CBPHS has been carried out for several years in China, it is necessary to evaluate the potential changes CBSPHS may bring in the DM patients. Therefore, the present study aimed to evaluate the awareness and knowledge about DR, as well as the associated risk factors, among DM populations registered in the CBPHS in China. The information will be very useful to better inform future policy-making regarding DR prevention and treatment.

Methods

Yueqing City is located in the east of Zhejiang Province, China, covering an area of 1286.90 square kilometers with a population of over 1 million. Yuecheng community and Nanyue community were selected to represent the urban and suburban areas of Yueqing City, respectively. Patients aged 18 years and above with a history of physician-diagnosed DM who were registered in the CBPHS were invited to participate in this study. In detail, there were 3156 DM patients in Yuecheng community and 1300 were randomly sampled. There were 923 DM patients in Nanyue community and all of them were invited to participate. The exclusion criteria included a DM history of less than 12 months and inability to cooperate with the interviewer. Questionnaires were administered by a trained community physician. The study was conducted between May and September 2017. Ethical approval was obtained from the Yueqing Hospital Ethical Review Board, and the study adhered to the Declaration of Helsinki. Written informed consent was received from all participants.

The study questionnaire was modified according to a previous study[8], consisting of three parts. The first part included questions assessing the patient's demographic and socioeconomic status, including age, gender, contact information, education level, monthly income, and information of medical insurance. The second part assessed the patient's medical history of DM, including the type, duration, diagnosis, and history of DM-related diseases. The third part assessed the patient's knowledge and

relevant behavior regarding DR, to include a knowledge section (7 questions about DR knowledge, e.g., Does DM affect eyes? How to know DM has affected your eyes?).

The primary outcome of the study was whether the participant knew that DM can affect the eyes, defined according to the question "Do you know diabetes mellitus can affect eyes? (yes or no)". Categorical and continuous outcomes were compared using Chi-squared tests and *t* tests, respectively. To assess the participants' knowledge about DR, a knowledge score was calculated based on the responses to 7 questions in the knowledge section, with 1 point awarded for a correct response and 0 points for an incorrect or uncertain answer. Multiple linear regression was performed to estimate the association between the knowledge score and other factors, such as the demographic and socioeconomic status and DM history. A P value <0.05 was considered to be statistically significant. All statistical analyses were performed using Stata 12.0 (StataCorp, College Station, TX, USA).

Results

A total of 1972 diabetic patients completed the questionnaires in the two communities, 1151 from Yuecheng community and 821 from Nanyue community. The mean (standard deviation) age was 65.2±10.8 years, and 45.7% were male. Among all study participants, 1219 patients (61.8%) knew that DM can affect the eyes. As shown in Table 1, subjects who knew that DM can affect the eyes were significantly younger than those who did not know (P<0.001). There were significant differences in education, income status and the insurance covering eye care between subjects who knew and those who did not know that DM can affect the eyes (P<0.001, P=0.028 and P<0.001, respectively). No significant difference existed regarding the insurance covering DM between patients in these two groups.

Table 1. Demographic and socioeconomic status of study participants

Factors	All （n=1972）	Did not know DM affects eyes (n=753)	Knew DM affects eyes (n=1219)	*P**

**Age (years), mean (SD)**	65.2 (10.8)	66.8 (10.7)	64.1 (10.7)	<0.001†
**Males, n (%)**	902 (45.7)	309 (41.1)	593 (48.7)	0.001‡
**Education, n (%)**				<0.001§
None	541 (27.4)	259 (34.4)	282 (23.1)	
Elementary school	641 (32.5)	241 (32.0)	400 (32.8)	
Junior high school	447 (22.7)	150 (19.9)	297 (24.4)	
High school	273 (13.8)	75 (9.96)	198 (16.2)	
College or above	70 (3.55)	28 (3.72)	42 (3.45)	
**Monthly family income (USD), n (%)**				0.028§
1-300	227 (11.5)	112 (14.9)	115 (9.43)	
301-450	327 (16.6)	124 (16.5)	203 (16.7)	
451-750	475 (24.1)	156 (20.7)	319 (26.2)	
751-1500	648 (32.9)	269 (35.7)	379 (31.1)	
>1500	295 (15.0)	92 (12.2)	203 (16.7)	
**Insurance covering DM, n (%)**				0.860‡
Self-pay	87 (4.41)	34 (4.52)	53 (4.35)	
Social health insurance	1,885 (95.6)	719 (95.5)	1,166 (95.7)	
**Insurance covering eye care, n (%)**				<0.001‡
Yes	423 (21.5)	156 (20.7)	267 (21.9)	
No	623 (31.6)	204 (27.1)	419 (34.4)	
Not sure	926 (47.0)	393 (52.2)	533 (43.7)	

SD: standard deviation, 1 USD = 6.67 RMB, DM: diabetes mellitus

* P values were for comparing participant characteristics between the two groups.

† *t* test was used.

‡ Chi-squared test was used.

§ Ordinal logistic regression was used.

As shown in Table 2, most patients in both groups had type 2 DM (96.9%). Subjects who knew that DM can affect the eyes had a higher fasting blood glucose and longer duration of diabetes compared to those who did not know (both P < 0.001). Approximately half of the subjects knew that they had DM through their physical check-up, 48.8% through presenting discomfort in the body and less than 1% through presenting discomfort in the eyes. Subjects who did not know that DM can affect the eyes had a lower proportion of using insulin and more concerns with using insulin (P<0.001 and P=0.002, respectively). In addition, more subjects who knew that DM can affect the eyes had a history of

hypertension (P=0.012), while the history of hypercholesterolemia was similar between the two groups.

Table 2. Medical history of diabetes among study participants by their knowledge of diabetes

	All （n=1972）	Did not know DM affect eyes (n=753)	Knew DM affect eyes (n=1219)	*P**
**DM type, n (%)**				0.051†
Type 1	61 (3.1)	16 (2.1)	45 (3.7)	
Type 2	1,911 (96.9)	737 (97.9)	1174 (96.3)	
**Fasting blood glucose (mmol/L) Mean (SD)**	7.00 (1.45)	6.86 (1.28)	7.08 (1.54)	0.001‡
**Years since DM diagnosed**	6.24 (4.38)	5.52 (3.96)	6.69 (4.57)	<0.001‡
**How to know you have DM, n (%)**				<0.001§
Physical check-up	999 (50.7)	423 (56.2)	576 (47.3)	
Discomfort in the body	963 (48.8)	326 (43.3)	637 (52.3)	
Discomfort in the eyes	10 (0.51)	4 (0.53)	6 (0.49)	
**Using insulin, n (%)**	184 (9.33)	46 (6.11)	138 (11.3)	<0.001†
**Concerns with using insulin, n (%)**				0.002¶
None	146 (7.56)	70 (9.50)	76 (6.37)	
Barely	525 (27.2)	167 (22.7)	358 (30.0)	
Some	848 (43.9)	303 (41.1)	545 (45.7)	
Substantial	411 (21.3)	197 (26.7)	214 (17.9)	
**History of hypertension, n (%)**	1,175 (59.6)	422 (56.0)	753 (61.8)	0.012†
**History of hypercholesterolemia, n (%)**				0.134†
No	1,335 (67.7)	519 (68.9)	816 (66.9)	
Yes	244 (12.4)	79 (10.5)	165 (13.5)	
Not sure	393 (19.9)	155 (20.6)	238 (19.5)	

SD: standard deviation

* P values were for comparing the participants' medical history between the two groups.

† Chi-squared test was used.

‡ *t* test was used.

§ Fisher's exact test was used.

¶ Ordinal logistic regression was used.

The prevalence of diagnosed DR was significantly higher among subjects who knew that DM can affect the eyes (P<0.001) (Table 3). Most subjects who knew that DM can affect the eyes obtained instructions

for regular eye examinations from physicians (57.8%). There were 1587 patients (80.9%) who had never undergone an eye examination, and only 178 patients (9.07%) underwent a yearly eye examination. The frequency of eye examinations was significantly different between the two groups (P<0.001). The main obstacle to having an eye examination was "vision is not affected" for all subjects, accounting for 41.1%. A lack of company was another important barrier to having an eye examination for the study participants in both groups. Regarding how the subjects knew that DM had affected their eye, subjects who knew through a fundus examination after pupil dilation only accounted for 5.98%; most subjects knew through discomfort in their eyes (43.1%). Only 29.5% of subjects thought that DM patients should have their eyes examined annually, and this percentage was significantly higher among subjects who knew that DM can affect the eyes. Additionally, 8.77% of subjects thought there was no need to have their eyes checked because of diabetes, and this percentage was significantly higher among subjects who did not know that DM can affect the eyes. Furthermore, people who did not know that DM can affect the eyes tended to be less anxious toward losing vision (P<0.001).

Table 3. History of ophthalmic care among diabetic patients by their knowledge of diabetes

	**All （n=1972）**	**Did not know DM affects eyes (n=753)**	**Knew DM affects eyes (n=1219)**	***P****
**Diagnosed with DR, n (%)**	95 (4.82)	14 (1.86)	81 (6.64)	<0.001†
**Told regular eye examination should be done, n (%)**				<0.001†
Never	613 (31.1)	434 (57.6)	179 (14.7)	
Yes, only by physician	966 (49.0)	261 (34.7)	705 (57.8)	
Yes, only by ophthalmologist	95 (4.82)	9 (1.20)	86 (7.05)	
Yes, by physician and ophthalmologist	298 (15.1)	49 (6.51)	249 (20.4)	
**Frequency of eye examinations, n (%)**				<0.001‡
Never	1,587 (80.9)	673 (90.2)	914 (75.2)	
More than 5 years	56 (2.85)	18 (2.41)	38 (3.13)	
Every 3 to 5 years	61 (3.11)	12 (1.61)	49 (4.03)	
Every 2 years	57 (2.91)	13 (1.74)	44 (3.62)	
Yearly	178 (9.07)	28 (3.75)	150 (12.3)	
More often than yearly	23 (1.17)	2 (0.27)	21 (1.73)	

**Told the time of next follow-up visit, among participants who ever had eye examination, n (%)**	246 (63.9)	29 (36.3)	217 (71.2)	<0.001†
**Obstacle to having an eye examination, n (%)**				<0.001§
No time	380 (19.3)	110 (14.6)	270 (22.2)	
No company	603 (30.6)	209 (27.8)	394 (32.3)	
Poor transportation	96 (4.87)	32 (4.25)	64 (5.25)	
Unreliable doctor	40 (2.03)	23 (3.05)	17 (1.39)	
No money	42 (2.13)	6 (0.80)	36 (2.95)	
Vision is not affected	811 (41.1)	373 (49.5)	438 (35.9)	
**How to know DM had affected eyes, n (%)**				<0.001†
Vision test	450 (22.8)	177 (23.5)	273 (22.4)	
Vision and ocular surface	139 (7.05)	43 (5.71)	96 (7.88)	
Discomfort in the eye, such as pain and blurry vision	850 (43.1)	280 (37.2)	570 (46.8)	
Fundus examination after pupil dilation	118 (5.98)	8 (1.06)	110 (9.02)	
Not sure	415 (21.0)	245 (32.5)	170 (14.0)	
**How often should diabetics have their eyes examined, n (%)**				<0.001†
Yearly	581 (29.5)	141 (18.7)	440 (36.1)	
Every 2 years	318 (16.1)	84 (11.2)	234 (19.2)	
3-5 years	91 (4.61)	36 (4.78)	55 (4.51)	
More than every 5 years	96 (4.87)	24 (3.19)	72 (5.91)	
Never	173 (8.77)	110 (14.6)	63 (5.17)	
Not sure	713 (36.2)	358 (47.5)	355 (29.1)	
**Concern about vision loss, n (%)**				<0.001§
Never	433 (22.0)	328 (43.6)	105 (8.61)	
Rarely	803 (40.7)	284 (37.7)	519 (42.6)	
Sometimes	562 (28.5)	117 (15.5)	445 (36.5)	
Often	130 (6.59)	19 (2.52)	111 (9.11)	
Very often	44 (2.23)	5 (0.66)	39 (3.20)	

* P values were for comparing the history of ophthalmic care between the two groups.

† Chi-squared test was used.

‡ Ordinal logistic regression was used.

§ Fisher's exact test was used.

The subjects' knowledge and attitudes about DR are shown in Table 4; the proportion of correct answers

to the other 6 questions regarding DR knowledge (besides DM can affect the eyes) ranged from 33.3% to 61.0%, with an average score of 3.65±2.47. The association between the knowledge score and other risk factors is shown in Table 5. In the multiple regression analysis, the knowledge score was significantly associated with age (P<0.001), education (P<0.001), income (P<0.001), history of hypertension (P=0.003), duration of DM (P<0.001), being told that regular examinations should be performed (P<0.001) and concern about vision loss (P<0.001).

Table 4. Knowledge about diabetic retinopathy among diabetic patients (n=1972)

	All	Correct	Correct ratio (%)
**Knowledge questions, n (%)**			
DM can affect eyes	1,219	1219	61.8
DM can cause blindness	999	999	50.7
DR is preventable	1,145	1145	58.1
DR is treatable	1,202	1202	61.0
Diabetic patients are more likely to get eye disease	1,105	1105	56.0
DR usually has early symptoms	659	659	33.4
Regular eye examinations are necessary	866	866	43.9

Table 5. Linear regression model for risk factors of participant's knowledge score about diabetic eye diseases (n=1972)

	Simple linear regression		Multiple linear regression	
	**□ (95% CI)**	***P***	**□ (95% CI)**	***P***
**Age (years)**	-0.03 (-0.04, 0.02)	<0.001	**-0.02 (-0.03, -0.01)**	**<0.001**
**Males**	0.32 (0.11, 0.54)	0.004	-0.001 (-0.19, 0.19)	0.991
**Education**				
None	Reference		Reference	
Elementary school	0.51 (0.23, 0.79)	<0.001	**0.54 (0.30, 0.78)**	**<0.001**
Junior high school	0.90 (0.59, 1.20)	<0.001	**0.97 (0.69, 1.24)**	**<0.001**
High school	1.08 (0.72, 1.43)	<0.001	**0.96 (0.64, 1.29)**	**<0.001**
College or above	1.18 (0.58, 1.79)	<0.001	**1.28 (0.76, 1.79)**	**<0.001**
**Monthly family income (USD)**				
1-300	Reference		Reference	
301-450	0.77 (0.35, 1.18)	<0.001	0.55 (0.21, 0.89)	0.001
451-750	0.87 (0.48, 1.25)	<0.001	0.46 (0.14, 0.78)	0.005

751-1500	0.44 (0.07, 0.81)	0.021	0.08 (-0.25, 0.40)	0.649
>1500	1.10 (0.68, 1.52)	<0.001	0.25 (-0.11, 0.61)	0.168
**Yuecheng community (Nanyue community as reference)**	0.19 (-0.03, 0.41)	0.090		
**Type 2 DM (type 1 as reference)**	-0.55 (-1.18, 0.08)	0.087		
**Fasting blood glucose (mmol/L)**	0.05 (-0.03, 0.12)	0.227		
**Using insulin (yes/no)**	0.94 (0.57, 1.31)	<0.001	0.28 (-0.03, 0.58)	0.078
**History of hypertension (yes/no)**	0.25 (0.02, 0.47)	0.030	**0.27 (0.09, 0.46)**	**0.003**
**Years since DM diagnosed**	0.07 (0.04, 0.09)	<0.001	**0.04 (0.02, 0.06)**	**<0.001**
**Diagnosed as DR (yes/no)**	1.08 (0.57, 1.58)	<0.001	0.31 (-0.11, 0.73)	0.149
**Used to have eye examinations (yes/no)**	0.96 (0.69, 1.23)	<0.001	0.14 (-0.10, 0.38)	0.244
**Told regular eye examinations should be done (never vs combined other options, never as reference)**	2.75 (2.55, 2.95)	<0.001	**2.07 (1.87, 2.28)**	**<0.001**
**Concern about vision loss (never vs combined other options, never as reference)**	2.56 (2.32, 2.80)	<0.001	**1.60 (1.37, 1.83)**	**<0.001**

1 USD = 6.67 RMB, DM: diabetes mellitus, DR: diabetic retinopathy

† All variables with P<0.05 in the simple regression analysis were included in the multiple regression analysis.

Discussion

In this study, we investigated the knowledge and attitudes regarding DR among adult DM patients registered in the CBPHS in eastern China via questionnaires, and found a generally low level of knowledge about DR in DM patients. Knowledge about DR was related to age, education, family income, hypertension, diabetic duration, concern about vision loss and being told that regular eye examinations should be performed. Our results showed that despite the local residents were given free regular blood glucose tests and other types of DM management through the existing chronic disease management system, but majority of patient still knew little of DR and its prevention. This finding reveals how to improve knowledge and attitude of DR within the chronic disease management system is the key to battle with this preventable blindness-causing disease.

In the current study, all the study participants were recruited from the local CBPHS system. We found that only 61.8% of the participants knew that DM can affect the eyes, and only 50.7% knew that DM can cause blindness. The proportion of correct answers to the other questions ranged from 33.4% to 61%, indicating a poor overall level of knowledge regarding DR among DM patients. This finding is similar to another population-based study in a suburban area (40.7%)[16], but lower than that reported in Shanghai (82.3%)[17]. However, we need to be aware that previous data were collected without the CBPHS; therefore, our findings did not show a positive impact of CBPHS on DR knowledge. There are large variabilities in the reported knowledge level about DR in the literature: 37% in Australia, 65% in the USA and 27% in India[18-20]. A hospital-based study in Zhejiang and a community-based study in Liaoning, China reported that 67% and 68% of the study subjects, respectively, were unaware that diabetes can affect the eyes[21, 22]. The differences among these studies could be partly explained by differences in health care systems, study populations and methods. Despite numerous health education programs on diabetes having been implemented, the IDF report shows the most DM patients still lack sufficient knowledge about the complications associated with diabetes[23]. Studies have reported that the most common complication known by DM patients is heart disease, followed by cerebrovascular and renal disease, while fewer people are aware that DM can also affect the eyes[24]. In our study, people who had been diagnosed with DR were more likely to know that DM can affect the eyes than those who had not. However, given 80.9% patients never underwent an eye examination in our study, the diagnosed rate of DR (4.82%) may be far less than the real DR prevalence. One study in urban China found that more than 90% of subjects with DR were unaware that they had been affected by this eye condition[25], and the Beijing Eye Study reported that only 15% of the study participants were aware of their DR[26].

We found that participants with a longer DM duration and higher fasting blood glucose had better awareness that DM can affect the eyes. This finding is of no surprise, as both the duration of DM and blood glucose levels are known risk factors for DM-related complications[25, 27]. Furthermore, people

with more severe DM and a longer duration of DM may have stronger initiatives and more opportunities to receive education regarding DM. Hypertension is another proven risk factor for DM and related complications[28], and people in our study with a history of hypertension also showed a better awareness that DM can affect the eyes. Even though dyslipidemia has been found to be a risk factor for DM[29], a history of hypercholesterolemia was not relevant to DR knowledge in our study. Patients with a better socio-economic background, including higher education level and income, had significantly better knowledge regarding DR compared to others. This is consistent with previous studies and could be due to better access to knowledge and a higher capability to understand[24, 30].

An annual dilated fundus examination is recommended for all patients with type 2 diabetes, but the reported awareness of the importance of routine check-ups is poor, even in developed countries[31]. In our study, only 29.5% of DM patients thought that they should have an annual eye examination, and the proportion was even lower for subjects who did not know that DM can affect the eyes (18.7% vs 36.1%). An awareness of regular examinations does not always lead to action. Only 10.2% of the study participants actually had yearly or more frequent eye examinations, which is much lower than that reported in United States (63%)[32], Switzerland (71%)[33] and Jordan (76%)[34]. No national DR screening program has been implemented in China. According to our study, 31.9% of the DM patients had never been told that a regular eye exam is necessary, and 80.9% of the DM patients had never had their eyes examined. The major barriers for eye examinations include a perception that their vision is not affected, followed by a lack of time and lack of company, suggesting that more educational programs and health support are needed to increase the service uptake among DM patients. Therefore, education about DR knowledge and the importance of regular eye exams should be enhanced in the CBPHS.

Physicians play an important role in imparting awareness and knowledge about DR. Our study found that most people knew about the necessity of regular eye examinations from a physician, and secondly,

from an ophthalmologist. Studies have also shown that physicians constitute the most important source of information in the knowledge gap for DM patients[35, 36]. In our study, only 6% of the participants knew that DM had affected their eye by a fundus examination after pupil dilation, and this proportion was as low as 1% for people who did not know that DM can affect the eyes. Most people knew that their eyes had been affected only after they experienced discomfort in the eye (43.1%), which could represent that the disease has evolved to later stages. Early detection and intervention are of vital importance to prevent sight-threatening and irreversible complications of DR. Thus, community-based and hospital-based educational programs, including posters, pamphlets, and screening camps, could be helpful for enhancing patients' awareness and knowledge to improve attitudes and practice. We believe that our study has highlighted the need for promoting knowledge regarding DR among DM patients, as well as shed light on potential barriers and interventions for policy-making in the future.

A limitation of this study concerns the representativeness of the study population, this study is based on the CBPHS and not strictly population-based. In addition, this study was cross-sectional in design, and further research is needed to investigate the short- and long-term health outcomes for DM patients with different levels of knowledge and attitudes toward DR.

In conclusion, with the existing chronic disease management system (free regular blood glucose tests and other types of DM management), the knowledge and attitudes regarding DR still haven't been improved. Routine ophthalmic screening and management of DR in DM patients should be emphasized, efforts should be directed toward health care promotions, and educational programs should be implemented for better patient outcomes. A more integrated and effective chronic disease management system is needed and should be improved gradually.

Data Availability

All relevant data are within the manuscript.

Funding

This study was supported partly by National Natural Science Foundation of China (81400381) and Zhejiang Medical and Health Science and Technology Project (2018269795).

Conflicts of Interest

The authors declare that they have no conflicts of interest.

Acknowledgments

We thank statistician Ling Jin, who provided the help for statistical analysis.

Authors' Contributions

Conceptualization: Xiang chen, Guoyi Zhou

Data curation: Yan Zheng, Qian Zhao, Yuedan Wu

Formal analysis: Fang Duan

Investigation: Qian Zhao, Ze Huang

Methodology: Fang Duan

Project administration: Xiang Chen

Resources: Guoyi Zhou

Supervision: Xiang Chen, Guoyi Zhou

Validation: Yuedan Wu

Writing-original draft: Fang Duan

Writing-review & editing: Xiang Chen, Guoyi Zhou

REFERENCES

1. Al-Lawati JA. Diabetes Mellitus: A Local and Global Public Health Emergency! Oman Med J. 2017;32(3):177-9. doi: 10.5001/omj.2017.34.

2. Cho NH, Shaw JE, Karuranga S, Huang Y, da Rocha Fernandes JD, Ohlrogge AW, et al. IDF Diabetes Atlas: Global estimates of diabetes prevalence for 2017 and projections for 2045. Diabetes Res Clin Pract. 2018;138:271-81. doi: 10.1016/j.diabres.2018.02.023.

3. Wang L, Gao P, Zhang M, Huang Z, Zhang D, Deng Q, et al. Prevalence and Ethnic Pattern of Diabetes

and Prediabetes in China in 2013. JAMA. 2017;317(24):2515-23. doi: 10.1001/jama.2017.7596.

4. Wang FH, Liang YB, Zhang F, Wang JJ, Wei WB, Tao QS, et al. Prevalence of diabetic retinopathy in rural China: the Handan Eye Study. Ophthalmology. 2009;116(3):461-7. doi: 10.1016/j.ophtha.2008.10.003.

5. Liu DP, Molyneaux L, Chua E, Wang YZ, Wu CR, Jing H, et al. Retinopathy in a Chinese population with type 2 diabetes: factors affecting the presence of this complication at diagnosis of diabetes. Diabetes Res Clin Pract. 2002;56(2):125-31.

6. Chen MS, Kao CS, Chang CJ, Wu TJ, Fu CC, Chen CJ, et al. Prevalence and risk factors of diabetic retinopathy among noninsulin-dependent diabetic subjects. Am J Ophthalmol. 1992;114(6):723-30.

7. Wang WQ, Ip TP, Lam KS. Changing prevalence of retinopathy in newly diagnosed non-insulin dependent diabetes mellitus patients in Hong Kong. Diabetes Res Clin Pract. 1998;39(3):185-91.

8. Wang D, Ding X, He M, Yan L, Kuang J, Geng Q, et al. Use of eye care services among diabetic patients in urban and rural China. Ophthalmology. 2010;117(9):1755-62. doi: 10.1016/j.ophtha.2010.01.019.

9. Almalki NR, Almalki TM, Alswat K. Diabetics Retinopathy Knowledge and Awareness Assessment among the Type 2 Diabetics. Open Access Maced J Med Sci. 2018;6(3):574-7. doi: 10.3889/oamjms.2018.121.

10. Vijan S, Hofer TP, Hayward RA. Cost-utility analysis of screening intervals for diabetic retinopathy in patients with type 2 diabetes mellitus. JAMA. 2000;283(7):889-96. doi: 10.1001/jama.283.7.889.

11. Thompson AC, Thompson MO, Young DL, Lin RC, Sanislo SR, Moshfeghi DM, et al. Barriers to Follow-Up and Strategies to Improve Adherence to Appointments for Care of Chronic Eye Diseases. Invest Ophthalmol Vis Sci. 2015;56(8):4324-31. doi: 10.1167/iovs.15-16444.

12. Duan F, Liu Y, Chen X, Congdon N, Zhang J, Chen Q, et al. Influencing factors on compliance of timely visits among patients with proliferative diabetic retinopathy in southern China: a qualitative study. BMJ Open. 2017;7(3):e013578. doi: 10.1136/bmjopen-2016-013578.

13. Lawrenson JG, Graham-Rowe E, Lorencatto F, Burr J, Bunce C, Francis JJ, et al. Interventions to increase attendance for diabetic retinopathy screening. Cochrane Database Syst Rev. 2018;1:CD012054. doi: 10.1002/14651858.CD012054.pub2.

14. Yip W, Hsiao W. Harnessing the privatisation of China's fragmented health-care delivery. Lancet. 2014;384(9945):805-18. doi: 10.1016/S0140-6736(14)61120-X.

15. Wang Z, Shi J, Wu Z, Xie H, Yu Y, Li P, et al. Changes in chronic disease management among community health centers (CHCs) in China: Has health reform improved CHC ability? Int J Health Plann Manage.

2017;32(3):317-28. doi: 10.1002/hpm.2433.

16. Hussain R, Rajesh B, Giridhar A, Gopalakrishnan M, Sadasivan S, James J, et al. Knowledge and awareness about diabetes mellitus and diabetic retinopathy in suburban population of a South Indian state and its practice among the patients with diabetes mellitus: A population-based study. Indian J Ophthalmol. 2016;64(4):272-6. doi: 10.4103/0301-4738.182937.

17. Yi Xiong L-PL, Yan Chen, Jie Zhao. Survey on the awareness of diabetic retinopathy among people with diabetes in the Songnan community of Shanghai. Int Eye Sci. 2015;15(7):1117-22.

18. Livingston PM, Wood CA, McCarty CA, Harper CA, Keeffe JE, Taylor HR. Awareness of diabetic retinopathy among people who attended a diabetic retinopathy screening program. Med J Aust. 1998;169(2):117.

19. Dandona R, Dandona L, John RK, McCarty CA, Rao GN. Awareness of eye diseases in an urban population in southern India. Bull World Health Organ. 2001;79(2):96-102.

20. Merz CN, Buse JB, Tuncer D, Twillman GB. Physician attitudes and practices and patient awareness of the cardiovascular complications of diabetes. J Am Coll Cardiol. 2002;40(10):1877-81.

21. Liu L, Chen L. Awareness of diabetic retinopathy is the key step for early prevention, diagnosis and treatment of this disease in China. Patient Educ Couns. 2014;94(2):284-5. doi: 10.1016/j.pec.2013.10.026.

22. Sapkota R, Chen Z, Zheng D, Pardhan S. The profile of sight-threatening diabetic retinopathy in patients attending a specialist eye clinic in Hangzhou, China. BMJ Open Ophthalmol. 2019;4(1):e000236. doi: 10.1136/bmjophth-2018-000236.

23. International Diabetes Federation Guideline Development G. Global guideline for type 2 diabetes. Diabetes Res Clin Pract. 2014;104(1):1-52. doi: 10.1016/j.diabres.2012.10.001.

24. Ullah F, Afridi AK, Rahim F, Ashfaq M, Khan S, Shabbier G, et al. Knowledge of Diabetic Complications in Patients with Diabetes Mellitus. J Ayub Med Coll Abbottabad. 2015;27(2):360-3.

25. Pan CW, Wang S, Qian DJ, Xu C, Song E. Prevalence, Awareness, and Risk Factors of Diabetic Retinopathy among Adults with Known Type 2 Diabetes Mellitus in an Urban Community in China. Ophthalmic Epidemiol. 2017;24(3):188-94. doi: 10.1080/09286586.2016.1264612.

26. Yang X, Deng Y, Gu H, Ren X, Lim A, Snellingen T, et al. Relationship of retinal vascular calibre and diabetic retinopathy in Chinese patients with type 2 diabetes mellitus: the Desheng Diabetic Eye Study. Br J Ophthalmol. 2016;100(10):1359-65. doi: 10.1136/bjophthalmol-2014-306078.

27. Xu J, Wei WB, Yuan MX, Yuan SY, Wan G, Zheng YY, et al. Prevalence and risk factors for diabetic retinopathy: the Beijing Communities Diabetes Study 6. Retina. 2012;32(2):322-9. doi: 10.1097/IAE.0b013e31821c4252.

28. Jee D, Lee WK, Kang S. Prevalence and risk factors for diabetic retinopathy: the Korea National Health and Nutrition Examination Survey 2008-2011. Invest Ophthalmol Vis Sci. 2013;54(10):6827-33. doi: 10.1167/iovs.13-12654.

29. Xu Y, Wang L, He J, Bi Y, Li M, Wang T, et al. Prevalence and control of diabetes in Chinese adults. JAMA. 2013;310(9):948-59. doi: 10.1001/jama.2013.168118.

30. AlHargan MH, AlBaker KM, AlFadhel AA, AlGhamdi MA, AlMuammar SM, AlDawood HA. Awareness, knowledge, and practices related to diabetic retinopathy among diabetic patients in primary healthcare centers at Riyadh, Saudi Arabia. J Family Med Prim Care. 2019;8(2):373-7. doi: 10.4103/jfmpc.jfmpc_422_18.

31. Bragge P, Gruen RL, Chau M, Forbes A, Taylor HR. Screening for presence or absence of diabetic retinopathy: a meta-analysis. Arch Ophthalmol. 2011;129(4):435-44. doi: 10.1001/archophthalmol.2010.319.

32. Eppley SE, Mansberger SL, Ramanathan S, Lowry EA. Characteristics Associated with Adherence to Annual Dilated Eye Examinations among US Patients with Diagnosed Diabetes. Ophthalmology. 2019. doi: 10.1016/j.ophtha.2019.05.033.

33. Konstantinidis L, Carron T, de Ancos E, Chinet L, Hagon-Traub I, Zuercher E, et al. Awareness and practices regarding eye diseases among patients with diabetes: a cross sectional analysis of the CoDiab-VD cohort. BMC Endocr Disord. 2017;17(1):56. doi: 10.1186/s12902-017-0206-2.

34. Bakkar MM, Haddad MF, Gammoh YS. Awareness of diabetic retinopathy among patients with type 2 diabetes mellitus in Jordan. Diabetes Metab Syndr Obes. 2017;10:435-41. doi: 10.2147/DMSO.S140841.

35. Ovenseri-Ogbomo GO, Abokyi S, Koffuor GA, Abokyi E. Knowledge of diabetes and its associated ocular manifestations by diabetic patients: A study at Korle-Bu Teaching Hospital, Ghana. Niger Med J. 2013;54(4):217-23. doi: 10.4103/0300-1652.119602.

36. Srinivasan NK, John D, Rebekah G, Kujur ES, Paul P, John SS. Diabetes and Diabetic Retinopathy: Knowledge, Attitude, Practice (KAP) among Diabetic Patients in A Tertiary Eye Care Centre. J Clin Diagn Res. 2017;11(7):NC01-NC7. doi: 10.7860/JCDR/2017/27027.10174.
